# Impact of pre‐therapy glioblastoma multiforme microenvironment on clinical response to autologous CMV‐specific T‐cell therapy

**DOI:** 10.1002/cti2.1088

**Published:** 2019-11-05

**Authors:** David G Walker, Reshma Shakya, Beth Morrison, Michelle A Neller, Katherine K Matthews, John Nicholls, Corey Smith, Rajiv Khanna

**Affiliations:** ^1^ Newro Foundation The Wesley Hospital Brisbane QLD Australia; ^2^ School of Medicine University of Queensland Brisbane QLD Australia; ^3^ QIMR Centre for Immunotherapy and Vaccine Development and Tumour Immunology Laboratory QIMR Berghofer Medical Research Institute Brisbane QLD Australia; ^4^ Department of Pathology Queen Mary Hospital The University of Hong Kong Hong Kong

**Keywords:** adoptive immunotherapy, cytomegalovirus (CMV), glioblastoma multiforme, tumor microenvironment

## Abstract

**Objectives:**

Clinical response to antibody‐based immunotherapies targeting checkpoint inhibitors is critically dependent on the tumor immune microenvironment (TIME). However, the precise impact of the TIME on adoptive cellular immunotherapy remains unexplored. Here we have conducted a long‐term follow‐up analysis of patients with recurrent glioblastoma multiforme (GBM) who were treated with autologous CMV‐specific T‐cell therapy to delineate the potential impact of the TIME on their clinical response.

**Methods:**

Multiplexed immunohistochemical analysis of CD3, PD‐L1 and Sox‐2 in GBM tissue biopsies obtained before autologous T‐cell therapy was carried out and correlated with long‐term survival of GBM patients adoptively treated with T‐cell therapy.

**Results:**

Tumor microenvironment analyses revealed that the pre‐treatment cellular composition of the tumor tissue may influence the subsequent response to adoptive T‐cell therapy. GBM patients who showed prolonged overall survival following T‐cell therapy had a significantly lower number of tumor‐infiltrating CD3^+^ T cells in recurrent tumors than that in patients with short‐term survival. Furthermore, long‐term surviving patients showed low or undetectable PD‐L1 expression in tumor cells in recurrent GBM biopsies.

**Conclusion:**

We hypothesise that lack of PD‐L1‐mediated immunosuppression in the TIME may allow efficient immune control following adoptive T‐cell therapy. Future studies combining anti‐PD‐L1 or genetically modified T cells with PD‐1 receptor knockdown could be considered to improve clinical responses in patients who have high PD‐L1 expression in their tumors.

## Graphical abstract

The precise role of tumor immune microenvironment (TIME) on clinical response to adoptive cellular immunotherapies remains largely unexplored. In this paper, we have analysed the impact of TIME on the clinical response of glioblastoma patients who showed either long‐term or short‐term overall survival following adoptive T‐cell therapy.

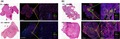

## Introduction

The outcomes for patients with glioblastoma multiforme (GBM) remain poor. The median survival of newly diagnosed patients receiving standard of care (surgical resection, radiotherapy and temozolomide chemotherapy) is 14.6 months.^1^ At recurrence, options for treatment are limited, and usually consideration is given to further surgery, reintroduction of first‐line chemotherapy (e.g. temozolomide) or second‐line chemotherapy (e.g. procarbazine, lomustine and vincristine, or bevacizumab), or further radiotherapy. However, the results of treatment for recurrent GBM are overwhelmingly poor and the median overall survival is approximately 30 weeks after the diagnosis.^2^

Immunotherapy has emerged as a powerful fourth pillar in cancer therapy, following the recent licensing of checkpoint blockade and adoptive cell therapies (ACT) for the treatment of melanoma, lymphoma and leukaemia amongst others. Despite the success of checkpoint blockade in many indications, results have been mixed in GBM, with recent phase III studies demonstrating no survival benefit following blockade of the PD1:PD‐L1 axis.^3^ One potential limitation on the efficacy of checkpoint inhibition is the low CD8^+^ T‐cell infiltrate detected in GBM.^4^ T‐cell immunotherapies have also been employed in early phase trials in GBM.^5^, ^6^ These trials have used non‐genetically modified T cells to target tumor‐associated antigens or viral antigens from human cytomegalovirus (CMV, which has a reported association with GBM), or have used genetically modified T cells expressing chimeric antigen receptors to target GBM‐related surface proteins. All of these approaches have shown some promise for the treatment of GBM.

The detection of CMV in GBM tissue, initially using immunohistochemistry but since validated using real‐time PCR and whole‐genome sequencing, has provided a platform to assess the use of CMV‐targeted approaches for the treatment of GBM. Current treatment approaches have focussed on the use of both anti‐viral and immune‐based therapies. We designed a CMV‐specific ACT approach for GBM based upon the administration of T cells expanded *in vitro* with CMV‐encoded peptide epitopes.^6^ We previously reported initial results and medium‐term follow‐up data for patients with recurrent GBM treated with anti‐CMV ACT.^6^ That trial commenced in 2009, and 12 patients received a minimum of two infusions of autologous CMV‐specific T cells as required per protocol. Here we report the long‐term follow‐up of these patients and attempt to characterise the features of long‐term survivors, including the impact of tumor‐associated immune contexture on outcome. Our observations suggest an association between long‐term survival following CMV‐specific ACT, low PD‐L1 expression in GBM tissue and a lack of CD3^+^ T‐cell infiltrate pre‐therapy. While these observations are from a small group of patients, we believe they can potentially offer valuable insights into this disease and guide the future development of GBM therapies.

## Results

### Patient characteristics and clinical outcome

Twenty‐two patients were screened for this trial, and the first patient was enrolled in November 2009. The first infusion for this patient was in January 2010, and the study closed in September 2014. Eligible patients were those with recurrent GBM (clinical and/or radiological evidence of recurrence), 18 years or older, who were able to be monitored and gave consent, had reasonable performance scores (ECOG 0–3), a life expectancy of at least 3 months, positive CMV serology and previously verifiable diagnosis of GBM.

CMV‐specific T cells for adoptive therapy were expanded from 15 patients, of which three patients were withdrawn prior to infusion because of progressive disease. Twelve patients received 2–4 T‐cell infusions, as per protocol, and are therefore included in the following analysis. No adverse events were detected that were deemed to be definitely related, probably related or possibly related to the investigational product. A summary of mild adverse events has been published previously.^6^

All patients received standard therapy at initial diagnosis, consisting of surgical resection, radiotherapy and chemotherapy (Table 1). The cohort of patients receiving 2 or more T‐cell infusions consisted of 4 women and 8 men. All patients receiving T cells had histologically confirmed glioblastoma multiforme following surgery for primary diagnosis or following resection for progressive disease (Table 2). Of the 12 patients included for analysis, enrolment occurred on average 3 (0.2–10.2) months following the most recent episode of disease progression. Disease progression immediately prior to study enrolment was determined by histological confirmation for 9 patients and MRI for the remaining 3 patients (Table 3).

**Table 1:** Participant histopathology for each surgical resection

	Initial surgery	Recurrent surgery		
		Surgery 1	Surgery 2	Surgery 3
GBM‐01	GBM	Recurrent GBM		
GBM‐02	Gliosarcoma			
GBM‐03	GBM	Anaplastic astrocytoma	Glioma	Necrotic tissue
GBM‐04	GBM	Recurrent GBM		
GBM‐06	Oligodendroglioma	Recurrent oligodendroglioma	GBM	
GBM‐07	GBM	GM		
GBM‐09	GBM	White matter degenerative changes		
GBM‐13	GBM	GBM		
GBM‐15	GBM			
GBM‐16	GBM			
GBM‐19	GBM	GBM	GBM	
GBM‐22	High‐grade astrocytoma	Oligoastrocytoma	GBM	

**Table 2:** Characteristics of patients with GBM and treatment history prior to T‐cell therapy

	Age at diagnosis	Gender	Histology preceding enrolment	Time from recurrence to enrolment (months)	Recurrence before T‐cell	Number of operations	Gliadel use	XRT/TMZ before T‐cell therapy	Additional Rx prior to T‐cell therapy
1	60.7	M	GBM	10.2	2	2	2	Yes	None
2	49.8	M	Gliosarcoma[Link]	0.3	1	1	0	Yes	Avastin
3	50.4	M	GBM	7.2	3	4	2	Yes	Etoposide, thalidomide
4	72.1	F	GBM	0.2	1	2	2	Yes	None
6	32.6	F	GBM	2.0	1	3	1	Yes	Avastin
7	54.1	M	GBM	1.3	1	2	0	Yes	Carboplatin
9	74.0	F	GBM	3.7	2	2	1	Yes	None
13	57.5	M	GBM	0.7	1	2	0	Yes	None
15	60.0	M	GBM[Link]	0.5	1	1	0	Yes	None
16	50.0	M	GBM[Link]	1.6	1	1	0	Yes	None
19	41.7	F	GBM	6.1	2	3	0	Yes	Carboplatin, lomustine, avastin
22	53.1	M	GBM	1.1	2	3	0	Yes	None

Confirmation of progression prior to T‐cell therapy on MRI.

**Table 3:** Clinical follow‐up of adoptive T‐cell therapy of patients with recurrent GBM

	Number of cells per infusion	Number of infusions	Time to progression after first infusion (months)	Treatment in addition to T cells	Follow‐up since first infusion (months)	Current status
1	3 × 10^7^	4		TMZ	108.7	Alive
2	2.8 × 10^7^	4	1.9	Avastin	10.8	Deceased
3	2.0 × 10^7^	3	4.4	TMZ, avastin, thalidomide	81.2	Deceased
4	2.9 × 10^7^	4		TMZ	33.2	Deceased[Link]
6	4 × 10^7^	4	1.2	Avastin, TMZ	5.0	Deceased
7	3.5 × 10^7^	2	0.7	TMZ	1.1	Deceased
9	2.5 × 10^7^	4	68.5	TMZ	76.3	Alive
13	3.2 × 10^7^	4	1.1	Avastin, CCNU	3.3	Deceased
15	2.5 × 10^7^	3	9.4		10.4	Deceased
16	4 × 10^7^	4	4.7	TMZ, surgery	15.1	Deceased
19	3 × 10^7^	3	7.7	Avastin, CCNU	10.6	Deceased
22	3 × 10^7^	4	6.9	Avastin	8.6	Deceased

Cause of death not related to GBM.

**Figure 1:**
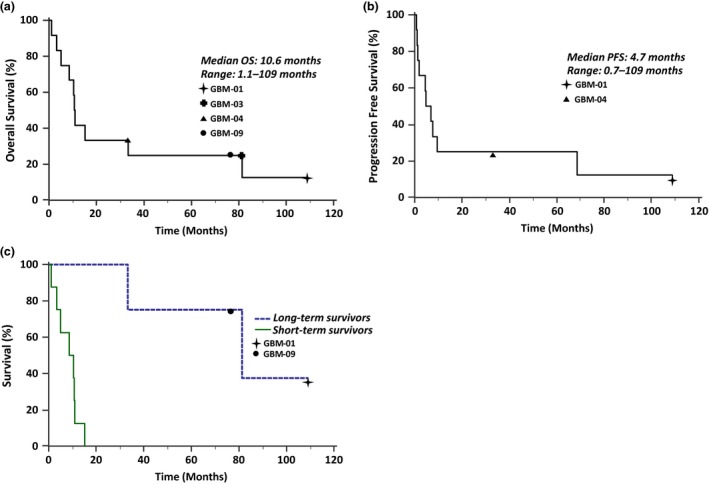
The Kaplan–Meier survival curves of GBM patients treated with autologous CMV‐specific T‐cell therapy. **(a)** Overall survival and **(b)** progression‐free survival of all 12 patients from date of first T‐cell infusion. **(c)** Overall survival of patients from first infusion, separated into long‐term survivors (*n* = 4) and short‐term survivors (*n* = 8) (*P* = 0.0025).

The survival data after the first infusion were previously reported by Schuessler *et. al.,* 6 and we have continued to follow this group of patients. The most recent survival and disease progression characteristics of the patient cohort are listed in Table 4. As of the end of February 2019, the median OS of the group from the date of first T‐cell infusion was 10.7 months (range 1.1–109 months) as shown in Figure 1a. The median PFS of the group from the date of first T‐cell infusion was 4.7 months (range 0.7–109 months) as shown in Figure 1b. These patients could be separated readily into two groups based on their overall survival (Figure 1c). Four long‐term survivors had a median overall survival from the time of their first T‐cell infusion of 81.2 months (range 33–109 months) compared to short‐term survivors (median 8.6 months, range 0.7–15.1 months).

**Table 4:** Characteristics of patients treated with CMV‐specific T‐cell therapy

Patient cohort (*n* = 12)	Range		Median
	Min	Max	
Age at consent (year)	38.3	75.1	54.6
Age at diagnosis (year)	32.6	74.0	52.2
OS following first infusion (months)	1.1	109.0	10.7
PFS following first infusion (months)	0.7	109.0	5.8

OS, overall survival; PFS, progression‐free survival.

### Clinical summary of long‐term survivors

**Figure 2:**
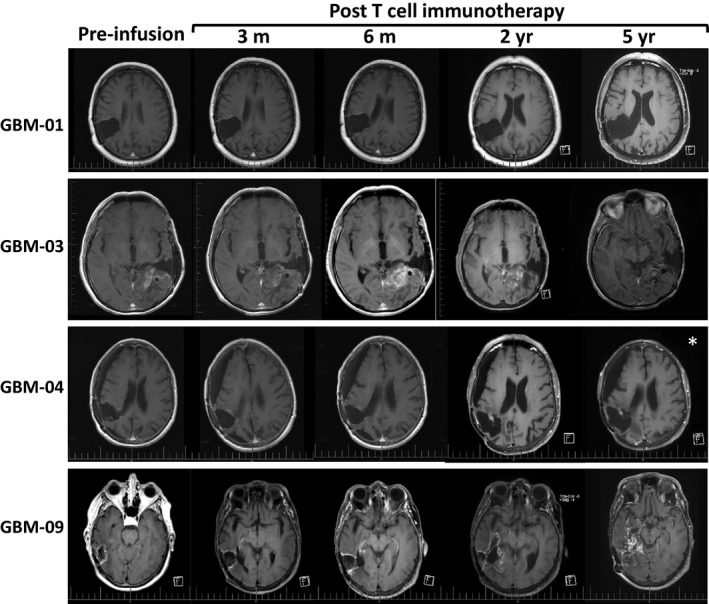
Representative T1^+^Gad axial MRI scans for long‐term survivors. In patient GBM‐03, enhancing tissue in the tumor bed became progressively more prominent over time, but there was little mass effect associated with this and the patient was clinically stable. Our interpretation therefore was that this enhancing tissue did not represent recurrent tumor. GBM‐04 showed no evidence of recurrent disease until the time of her untimely death 2.5 years after T‐cell infusion (*) from a basilar artery aneurysm (unrelated to adoptive T‐cell therapy).

*Patient 1, GBM 01*: This man was 61 years old when he presented with left‐sided weakness in February 2008. He underwent a resection of a right‐sided parietal tumor and carmustine wafers were placed during this initial operation. The histology supported a diagnosis of GBM. Tumor cells showed no evidence of isocitrate dehydrogenase 1 (IDH1) mutation and were positive for O6‐methylguanine DNA methyltransferase (MGMT) methylation status. Standard therapy (6 weeks of radiotherapy with concomitant temozolomide and subsequent monthly temozolomide treatment) continued up until January 2009, when on a routine follow‐up MRI scan, local tumor recurrence was identified. Further resection with carmustine wafer placement was undertaken. Monthly temozolomide treatment continued and in May 2009, a follow‐up MRI scan showed likely local tumor progression (Figure 2). This patient was the first to be enrolled into the CMV‐specific ACT trial in November 2009, approximately 10 months following most recent occurrence of disease progression. During and after the ACT infusions, he continued with monthly temozolomide. The last of his four infusions was in March 2010, and MRI scans showed a complete radiological response. This patient remains well, with serial disease surveillance MRI scans at 3 months, 6 months, 2 years and 5 years showing no evidence of tumor recurrence (Figure 2).

*Patient 3, GBM 03:* This man initially presented at age 50 in 2006, following a seizure. A left temporal GBM was resected and carmustine wafers were inserted. Tumor cells were positive for IDH1 mutation and positive for MGMT methylation status. Standard post‐operative radiotherapy and temozolomide chemotherapy were given. The tumor recurred locally 7 months later (in 2007) and a second resection was undertaken. Further radiological recurrences occurred in February and June of 2010, which led to resection on both occasions (Figure 2). After about 7 months following further disease progression, this patient was enrolled in this clinical trial. Three infusions of CMV‐specific T cells were given, starting in November 2010. The patient continued to receive daily low‐dose temozolomide (100 mg) between 2008 and 2015, and also received bevacizumab chemotherapy at regular intervals between 2011 and 2015. Progressive disease resulted in death in August 2017.

*Patient 4, GBM 04:* This 72‐year‐old female patient presented with a seizure and left‐sided weakness in June 2009. A right parietal tumor, consistent with GBM, was seen on MRI; the tumor was resected and carmustine wafers were inserted. GBM was confirmed by histopathology and standard chemoradiation followed. Tumor cells were negative for IDH mutation and positive for MGMT methylation status. Radiological evidence of recurrence was observed 15 months after the initial diagnosis, and resection with carmustine wafers was again undertaken. The patient then entered the trial in less than 1 month following tumor progression (Figure 2), with continuation of temozolomide. Subsequent monitoring based on MRI scans showed no evidence of tumor recurrence following four CMV‐specific T‐cell infusions (Figure 2). However, the patient suffered from progressive bulbar palsy, secondary to a giant fusiform aneurysm of the basilar artery, which led to her death in June 2013. There was no radiological evidence of tumor recurrence at this time.

*Patient 9, GBM 09:* This 74‐year‐old female patient presented with visual field loss and headaches in May 2011. A right occipitoparietal GBM was resected and standard therapy ensued. Tumor cells were negative for IDH mutation and positive for MGMT methylation status. Radiological evidence of recurrence was seen in January 2012, and resection with carmustine wafer insertion occurred. The patient then entered the trial, and in the interim, temozolomide was given at different time points. She has largely remained well and continues to live at home, but her level of functioning has slowly deteriorated. Multiple MRI scans have shown enhancing tissue in the region of the previously treated tumor (Figure 2). The degree of enhancing tissue has slowly increased between 2 and 5 years post‐CMV‐specific T‐cell therapy (Figure 2). Whether the changes on scans represent discrete tumor progression has been unclear. This patient remains well despite the confirmation of tumor progression at 5 years and 8 months post‐infusion.

### Quantitative multiplexed immunohistochemical assessment

**Figure 3:**
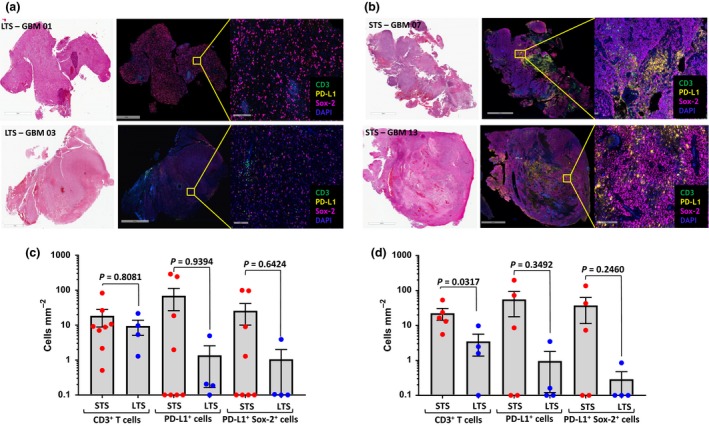
Multiplexed immunohistochemical analysis of CD3, PD‐L1 and Sox‐2 in GBM tissue biopsies obtained before autologous T‐cell therapy. Representative haematoxylin and eosin stained (left panels) and multiplexed immunohistology staining of CD3, PD‐L1 and Sox‐2 in tumor biopsies (right panels) of **(a)** long‐term (GBM 01 and GBM 03) and **(b)** short‐term (GBM 07 and GBM 13) surviving patients, respectively. **(c)** Overall summary of CD3, PD‐L1 and Sox‐2 expression in tumor biopsies of primary GBM patients. **(d)** Overall summary of CD3, PD‐L1 and Sox‐2 expression in tumor biopsies of recurrent GBM patients. LTS: long‐term survivors; STS: short‐term survivors. Each symbol represents data from individual patients. Bar represents median values.

We sought to explore the immunological correlates that may be associated with the long‐term survival of these four patients. As previously reported,^6^ our initial analyses of peripheral blood immunity provided limited evidence for changes in cellular immunity that could be associated with an improved outcome in these patients. Recent observations in other immunotherapeutic settings have shown that the tumor immune microenvironment (TIME) can have a significant impact on efficacy. We therefore sought to investigate the TIME in our long‐term survivors and compare the results with patients who had short‐term survival. Recurrent tumors from nine patients (all four long‐term survivors and five of the short‐term survivors) were available for detailed immunolabelling, while primary tumors were available from 12 patients (all four long‐term survivors and eight of the short‐term survivors). Tumor sections were assessed for the presence of T cells (via CD3 staining) and the expression of PD‐L1. Sox‐2 was used as a marker for glioma cells.^7^ Representative images of recurrent GBM tissue sections from two long‐term survivors (Figure 3a) and two short‐term survivors (Figure 3b) are shown.

Detailed analysis using multiplexed immunofluorescence and automated cell counting revealed differences in the TIME of long‐term and short‐term survivors. Long‐term survivors displayed significantly reduced number of CD3^+^ T cells in comparison to short‐term survivors in recurrent tumor sections (Figure 3d), while no significant difference was evident in the primary tumor sections (Figure 3c). Interestingly, we also saw a trend towards a decrease in the density of PD‐L1‐expressing cells in the recurrent tumor tissue of long‐term survivors compared with short‐term survivors (Figure 3d). Although this difference did not reach statistical significance because of the small number of patients in each group (*P* = 0.3492), none of the long‐term recurrent GBM survivors displayed a density of greater than ten PD‐L1 expressing cells mm^−2^. This trend was also evident in the primary tumor sections (Figure 3c), and the difference was predominantly associated with the differential expression of PD‐L1 on Sox‐2‐expressing tumor cells, although this difference was also not significant (*P* = 0.6424). PD‐L1 expression was also observed in Sox‐2‐negative (non‐tumor) cells; however, it was not present on tumor‐infiltrating CD3^+^ T cells. While these observations are preliminary and rely upon a small cohort of patients in whom overall survival could be influenced by other factors, they do suggest that the TIME may have an impact on the long‐term response of recurrent GBM patients to cellular immunotherapy.

## Discussion

Given that standard therapy for malignant glioma almost always fails, there has been a drive to develop more effective treatments over many years. This has included manipulation of the immune system, such as the use of dendritic cell vaccines,^8^ peptide vaccines,^9^ heat‐shock protein vaccines^10^ and ACT.^11^ Overall, the results have been mixed, but some promising trends have emerged. Cobbs *et al.* demonstrated the presence of CMV proteins in GBM specimens.^12^ Whilst the potential role of CMV in glioma cells is uncertain, these ‘'foreign’ antigens can serve as potential targets for immune therapies.^13^ We previously reported our initial experience in the use of CMV‐specific ACT for patients with recurrent GBM,^6^ and the current paper provides updated data for that cohort. It has become clear, over a long period of time, that a subset of patients in the study group have exhibited extraordinary survival times. Based on the multiplexed immunofluorescence studies that are presented here, it appears that CMV‐specific ACT is effective in significantly prolonging survival in patients with recurrent GBM when there are a few PD‐L1^+^ cells in the tumor tissue prior to therapy. The results imply that a possible mechanism of failure of CMV‐specific ACT as a stand‐alone treatment can be attributed to the presence of PD‐L1^+^ cells in the tumor tissue. However, it should be noted that low PD‐L1 expression was also observed in a proportion of short‐term survivors, suggesting that other immune evasion mechanisms are potentially impacting the survival of these patients.

While long‐term survival following diagnosis of relapsed GBM is exceedingly rare, a number of molecular characteristics have been associated with improved overall survival in GBM patients, including the presence of IDH mutation and MGMT promoter methylation. We did not notice any link between IDH mutations and long‐term surviving patients. The presence of IDH1 mutation is almost always observed in younger patients, and in those with secondary GBM. Older patients, presenting with de novo primary GBM, do not as a rule harbour IDH1 mutations in their tumors. It is of interest in our small cohort that the longer survival rates have been observed in older patients, and three of the four patients showed no IDH1 mutations, which would normally imply a worse prognosis. The average survival of patients with IDH1‐mutant GBM is 31 months compared to 15 months in IDH1 wild‐type patients after standard therapy,^14^ and the survival in our small group of patients was significantly longer than this. Similarly, MGMT promoter methylation has been associated with improved response to chemotherapy and therefore survival. Since hypermethylated phenotypes are associated with IDH1‐mutant tumors (and hence younger patients), MGMT promoter methylation is also unlikely to have a dramatic impact on the current cohort of long‐term survivors. Another potential factor that could impact upon overall survival following T‐cell therapy is ongoing standard‐of‐care treatment in patients. While most patients were treated with standard temozolomide, a number of patients including patient 03 also received bevacizumab. However, it should be noted that it is highly unlikely that bevacizumab (Avastin) has strongly influenced the long‐term survival on its own, as this drug has not been shown to significantly increase survival in patients with GBM.^3^ In addition, other patients in the study who also received bevacizumab did not show long‐term survival.

PD‐L1 expression in GBM tissue has previously been analysed by others. Nduom *et al.* 15 found that the percentage of PD‐L1^+^ cells was generally low in glioma tissue (average 2.77%) and the expression of PD‐L1 and PD‐1 were negative prognosticators of outcome. The authors reported that the median overall survival for newly diagnosed patients was 11.4 months or 14.9 months in tumors that showed high or low PD‐L1 expression, respectively. As we observed a median overall survival of 81.2 months in our cohort of long‐term survivors, it is possible that CMV‐specific ACT contributed to tumor control in these patients. In recent years, immune checkpoint inhibitors have been used in a variety of cancers, including melanoma,^16^ Hodgkin’s lymphoma^17^ and lung cancer.^18^ However, in a recent trial of PD‐1 inhibition in recurrent GBM, only a small proportion of patients showed a response to this treatment.^3^ PD‐1 blockade is thought to act by disrupting the inhibition of PD‐1^+^ T cells by inhibitory ligands (i.e. PD‐L1), enabling cytotoxic T‐cell‐mediated tumor cell attack. Zhao *et al*.^19^ analysed a cohort of GBM patients treated with anti‐PD1 therapy (nivolumab) and compared responders to non‐responders. Responders were classified by tumor stabilisation or shrinkage via MRI, or few to no tumor cells within biopsies from surgery post‐therapy. Non‐responders, who had reduced long‐term survival compared with responders, had enrichment of phosphatase and tensin homologue (PTEN) mutations in their tumors, and this corresponded with a lack of T‐cell infiltration. PTEN mutations are rare in IDH‐mutant GBM, but occur in approximately 25% of IDH wild‐type tumors. Cloughesy *et al.* 20 recently reported a small randomised study comparing the outcomes of patients with resectable recurrent GBM who received neoadjuvant (i.e. starting before surgical resection) and post‐surgical pembrolizumab, to those of patients who only received post‐surgery pembrolizumab. Patients receiving neoadjuvant PD‐1 blockade had a significant survival advantage (417 vs. 228 days overall survival, *P* = 0.04). By analysing tumor specimens from surgery, the investigators found there was activation of tumor‐infiltrating lymphocytes in patients who received pre‐surgery pembrolizumab. The authors proposed that this led to systemic priming of T cells and therefore the effective production of anti‐tumor T‐cell clones. Similar results have been found by others using neoadjuvant nivolumab.^21^ In the latter study, neoadjuvant nivolumab did not appear to result in prolonged survival of the treated patients; however, their results suggested that combined immunotherapies may be more efficacious.^22^

Our preliminary studies in patients from our phase I study suggested that there are differences between patients who displayed long‐term survival compared with those who did not. In particular, the pre‐treatment cellular makeup appeared to have an impact on long‐term survival following ACT. The long‐term survival patients displayed lower numbers of PD‐L1^+^ cells within tumor specimens compared to the short‐term survivors. This was especially true when examining the frequency and density of PD‐L1^+^Sox‐2^+^ and PD‐L1^+^ cells. One potential mechanism by which this could limit the efficacy of ACT is the inactivation of the infused CMV‐specific T cells when they encounter the inhibitory ligand, PD‐L1, within the tumor. PD‐L1 in this context could be expressed on tumor cells, regulatory T cells or antigen‐presenting cells. Sox‐2 is a stem cell transcription factor needed to induce and maintain the stemness of GBM cells.^23^, ^24^ Therefore, the apparent biggest difference between long‐term and short‐term survivors in our study was a higher proportion and density of PD‐L1^+^ tumor cells in short‐term survivors.

Based on the results of our trial, in which long‐term survival following CMV‐specific ACT was associated with a lower density of PD‐L1^+^ cells, a logical next step would be to combine this therapy with another treatment aimed at blocking the PD‐1/PD‐L1 pathway. For instance, neoadjuvant PD‐L1 blockade (e.g. using atezolizumab) before giving ACT may be effective. An alternative strategy, given the results of Cloughesy *et al*.,^20^ would be to commence PD‐1 inhibition prior to surgery, continue PD‐L1 blockade after surgery if tumors showed expression of PD‐L1 and then administer ACT. The T cells could perhaps be engineered to withstand PD‐1‐mediated inhibition, as recently reported,^25^ or this receptor could be blocked *in vitro* before delivery. Together with the results of others, our trial gives hope that effective treatments for GBM are on the horizon.

## Methods

### Patients and samples

This study was conducted in accordance with the Declaration of Helsinki principles after approval by independent ethics committees, with written informed consent from all patients prior to specimen testing. Tumor samples from patients with recurrent GBM (*n* = 11), who received autologous CMV‐specific T‐cell therapy during our phase I clinical trial (ACTRN12609000338268) between 2009 and 2014, were used for this study. Formalin‐fixed paraffin‐embedded tissue specimens were obtained prior to the commencement of T‐cell therapy, and 4‐μm‐thick serial sections were generated for subsequent multiplexed immunohistochemistry. The following clinicopathological parameters were collected for statistical analysis: patient age at diagnosis, date of surgery and recurrence, gender, histological grade of tumor, prior chemotherapy and/or radiotherapy, number and date of infusions, progression‐free survival (PFS) from time of diagnosis, overall survival (OS) from time of diagnosis, PFS following T‐cell infusion and OS following T‐cell infusion. OS following T‐cell infusion was defined as the time from the first T‐cell infusion to the date of death and PFS following T‐cell Infusion was defined as time from the first T‐cell infusion to the date of progression, as determined by MRI scans.

### Fluorescent multiplexed immunohistochemical staining

Sections cut from formalin‐fixed paraffin‐embedded blocks were dewaxed with xylene and rehydrated by passage through a graded series of ethanol washes (100%, 90%, 70% and 50%), before immersing in distilled water. Endogenous peroxidase was blocked by incubating the sections with 0.5% H_2_O_2_ for 5 min. Heat‐induced antigen retrieval was performed by microwaving the samples for 2 min 20 sec with 100% power, followed by 20% power for 15 min, and the slides were then cooled at room temperature for 20 min. Sections were then incubated with Background Sniper blocking solution (Biocare Medical, Concord, CA) supplemented with 2% BSA for 15 min at room temperature. Slides were stained with the following primary antibodies for 30 min at room temperature: CD3 (Dako A0452, diluted 1/6000 in Dako Target Retrieval Solution pH 9), PD‐L1 (Cell Signalling Technology E1L3N, diluted 1/100 in Dako Target Retrieval Solution pH 9) and Sox‐2 (Abcam ab92494, diluted 1/6000, Dako Target Retrieval Solution pH 9). The immunofluorescent signal was detected using the Opal 7‐color automation IHC kit (Perkin Elmer, Waltham, MA) according to the manufacturer’s instructions. Antibodies were stripped by heating the slides in citrate buffer, pH 6, to allow subsequent staining of other antigens. Slides were then counterstained with DAPI for 5 min and mounted with Dako Fluorescent Mounting Medium (DAKO, Glostrup, Denmark).

### Digital image acquisition and automated quantification

Whole slide images were scanned at an absolute magnification of 20 × on an Aperio ScanScope FL system (Leica Biosystems, Nussloch, Germany). Image analysis was performed using the HALO platform (Indica Labs, Corrales, NM). First, haematoxylin and eosin stained slides from all the patients were reviewed by a certified pathologist and the acceptable tumor regions were marked using an Aperio ImageScope (Aperio Technologies, Inc., California). Using HALO software, an algorithm was designed based on pattern recognition that quantified immune cells within pathologist‐selected tumor areas of multiplexed tissue. Those areas lacking tumor cells, containing mainly necrotic cells or false‐positive fluorescence signals, were omitted from the analysis. Individual cells within marked regions were segmented into nuclei, cytoplasm and membrane. The DAPI signal was used to identify cell nuclei. Sox‐2 detected with Opal 690 was used as a glioma marker. The algorithms were able to recognise CD3‐, PD‐L1‐ and Sox‐2‐stained cells, and the densities (cells mm^‐2^) of positive cells within the tumor region were calculated. The densities of CD3^+^, PD‐L1^+^ and Sox‐2^+^ cells were correlated with clinicopathological parameters.

### Statistical analysis

OS and PFS following T‐cell infusion were summarised by Kaplan–Meier curves. Overall survival following T‐cell infusion was defined as the time (in months) to death from the date of first T‐cell infusion and PFS following T‐cell infusion was defined as the time (in months) to diagnosis of further disease progression from the date of first T‐cell infusion. Statistical analysis was undertaken in MedCalc Software Version 18.11.6.

## Conflict of interest

CS and RK receive research and consultancy funding from Atara Biotherapeutics Inc. RK is also appointed as an advisor on the Atara Biotherapeutics Scientific Advisory Board. CS and RK hold international patents or patent applications that cover CMV epitope sequences and their use in adoptive immunotherapy.

## Grant support

This study was supported by the National Health and Medical Research Council of Australia (NH&MRC) Australia (Project ID: APP1020325) and the Rio‐Tinto Ride to Conquer Cancer Flagship Program. RK is supported by a Senior Principal Research Fellowship from NH&MRC.
